# Evaluation of skin cancer resection guide using hyper-realistic in-vitro phantom fabricated by 3D printing

**DOI:** 10.1038/s41598-021-88287-4

**Published:** 2021-04-26

**Authors:** Junhyeok Ock, Taehun Kim, Sangwook Lee, Tae Seong Yang, Minji kim, Wooshik Jeong, Jongwoo Choi, Namkug Kim

**Affiliations:** 1 Department of Convergence Medicine, Asan Medical Institute of Convergence Science and Technology, University of Ulsan College of Medicine, Asan Medical Center, 88 Olympic-Ro 43-Gil Songpa-Gu, Seoul, South Korea; 2 Department of Plastic and Reconstructive Surgery, University of Ulsan College of Medicine, Asan Medical Center, 88 Olympic-Ro 43-Gil Songpa-Gu, Seoul, South Korea; 3 Department of Radiology, University of Ulsan College of Medicine, Asan Medical Center, 388-1 Pungnap2-dong, Songpa-gu, Seoul, South Korea; 4 Department of Plastic and Reconstructive Surgery, Ajou University Hospital, School of Medicine, Suwon, South Korea; 5 ANYMEDI Inc., Seoul, South Korea

**Keywords:** Skin cancer, Biomarkers

## Abstract

Skin cancer usually occurs in the facial area relatively exposed to sunlight. Medical imaging can confirm the invasiveness and metastasis of skin cancer, which is used to establish a surgical plan. However, there is no method of directly marking this information on the patient's skin in the operating room. We evaluated a skin cancer resection guide that marks resection areas including safety margins on the patient's skin based on medical images and in-vitro phantom fabricated via 3D printing. The in-vitro phantom, which includes the skull, skin, and five different cancer locations was designed and fabricated based on a CT image of a patient. Skin cancer resection guides were designed using a CT image of an in-vitro phantom, with a safety margin, and four injection points at each cancer. The guide was used to insert 16 cc intravenous catheters into each cancer of the phantom, which was rescanned by CT. The catheter insertion point and angle were evaluated. The accuracy of the insertion points was 2.09 ± 1.06 mm and cosine similarities was 0.980 ± 0.020. In conclusion, skin cancer resection guides were fabricated to mark surgical plans on the patient's skin in the operating room. They demonstrated reasonable accuracies in actual clinical settings.

## Introduction

Environmental pollution causes depletion of the ozone layer, which increases UV exposure. Excessive UV exposure causes skin cancer by directly damaging skin cells or via abnormal immunological functions. Consequently, the number of skin cancer cases is increasing worldwide^1,3^. Skin cancer is mainly diagnosed by basal cell carcinoma (BCC), squamous cell carcinoma (SCC), and malignant melanoma^2^. It mainly occurs on the face and neck, which are constantly exposed to sunlight. The face, nose, cheeks, lower eyelids, and forehead are more common^3^. Skin cancer is mainly diagnosed in western countries, including the United States, but the diagnosis rate has also recently increased in South Korea^4^. Generally, it is examined using medical images obtained via methods such as computerized tomography (CT) and magnetic resonance (MR), and the resection area is determined by a biopsy report. The type and size of the lesion and resection area are extracted using surgical treatment^5^. The formation of skin cancer is often deep and wide along the neural tube, cartilage, and embryonic fusion planes, which results in a longer operating time than expected. To overcome this, Mohs Micrographic surgery, in which the resection of skin cancer is checked in real-time through a microscope, was devised. In this method, the resection area is minimized, and the skin cancer cure rate is high; but operating time is increased and high cost^6^. Although medical imaging methods such as CT or MRI confirm the extent of disease invasion and metastasis and help establish a surgical plan, no method directly marks this information on the patient's skin in the operating room. This study aimed to devise a method based on 3D printing technology and medical images, which accurately marks the incision area of skin cancer, and minimizes operating time. 3D printing technology is widely used in the medical field^7^. It is used in various applications such as patient-specific surgical guides, simulators for rehearsal surgery, patient education, and patient-specific implants^8–11^. Several previous studies have used 3D printing technology to fabricate a simulator with complex anatomies such as a difficult airway intubation simulator^9^, A compliant aortic simulator^12^, a gastric simulator^13^, and a thyroid cancer phantom^14^. These simulators may provide better training opportunities for the trainee and allow for in-vitro experiments. In addition, 3D printing technology based on medical images is used to fabricate various kinds of patient-specific surgical guides such as breast cancer resection guide^15^, talocalcaneal coalition resection guide^16^, and implant drilling guide^17^. These surgical guides can deliver accurate surgical plans in the operating room. In this study, a hyper-realistic in-vitro phantom, which includes the skull, skin, and five different cancer locations was designed and fabricated based on a CT image of a patient. In addition, we evaluated the accuracy of a skin cancer resection guide with the in-vitro phantom.

## Results

### Fabrication of in-vitro phantom and surgical guide

**Figure 1:**
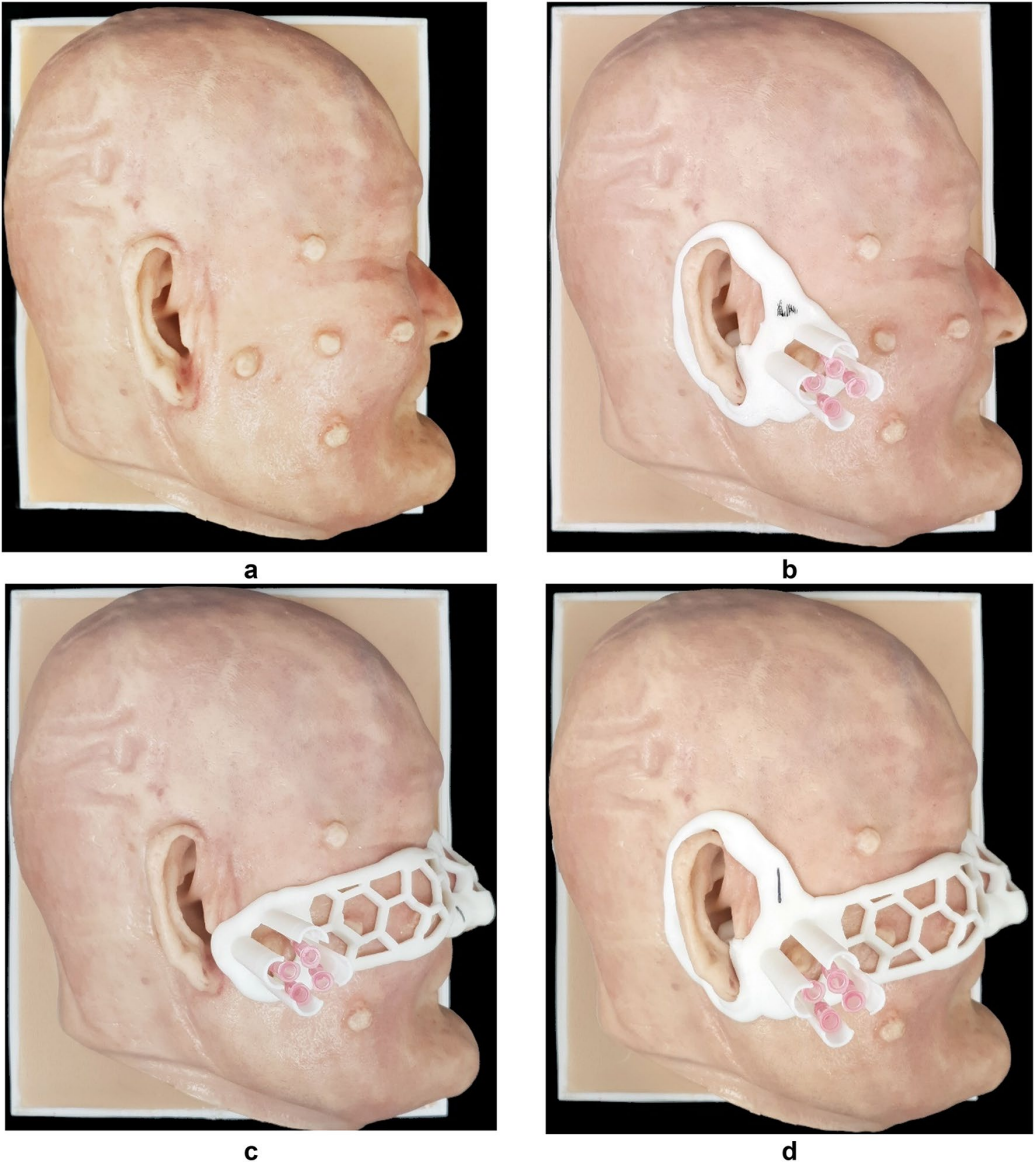
Fabricated in-vitro phantom and inserted 16 cc intravenous (IV) catheter using the skin cancer resection guide. (**a**) Fabricated in-vitro phantom. (**b**) Inserted 16-cc IV catheters into in-vitro phantom using skin cancer resection guide attached at the ear. (**c**) Inserted 16-cc IV catheters into in-vitro phantom using skin cancer resection guide attached at the nose. (**d**) Inserted 16-cc IV catheters into in-vitro phantom using skin cancer resection guide attached at both the ear and nose.

The skin molder was fabricated using color-jet printing (CJP) with plaster (ProJet 460,3D Systems, USA), based on the size of the model and separation with silicone. The skull was fabricated using CJP considering the strength of the actual bone, the size of the model, and Hounsfield unit (HU) value. The cancer was fabricated by stereolithography apparatus (SLA) with clear resin (Form2, Formlabs, USA), considering the relatively low price and HU value. The cancer was put on the skin molder and silicone (Dragon-skin-fx-pro, Smooth-on, USA) was coated twice using a brush. After the completion of the silicone coating, the skull was combined with the skin molder, and silicone was injected. The skin molder was broken, and the skin removed after 1 day, for the silicone to cure sufficiently (Fig. 1a). The skin cancer resection guide was modeled based on CT images of the in-vitro phantom and fabricated using fused deposition modeling (FDM) with Z-ultrat (M200, Zortrax, Poland) (Fig. 1b–d).

### Evaluation of skin cancer resection guide

**Figure 2:**
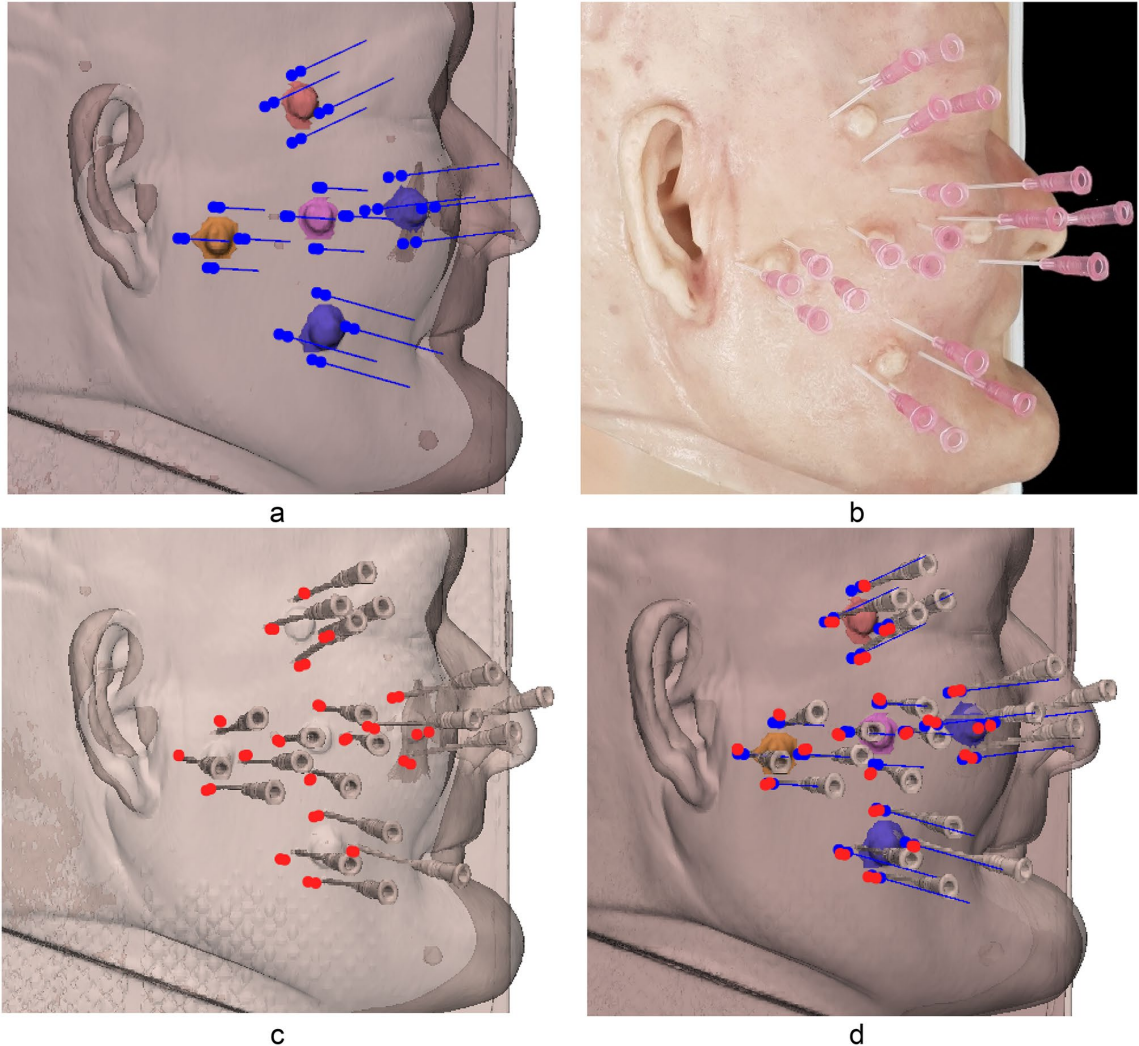
Visualization of the planned resection point using in-vitro phantom CT image and inserted 16 cc intravenous (IV) catheter into in-vitro phantom using skin cancer resection guide and matching planned points with actual inserted points. (**a**) Planned insertion points and line. (**b**) Inserted 16 cc IV catheters into in-vitro phantom using skin cancer resection guide. (**c**) Actual insertion points of 16 cc IV catheter. (**d**) Matching planned points with actual inserted points.

**Figure 3:**
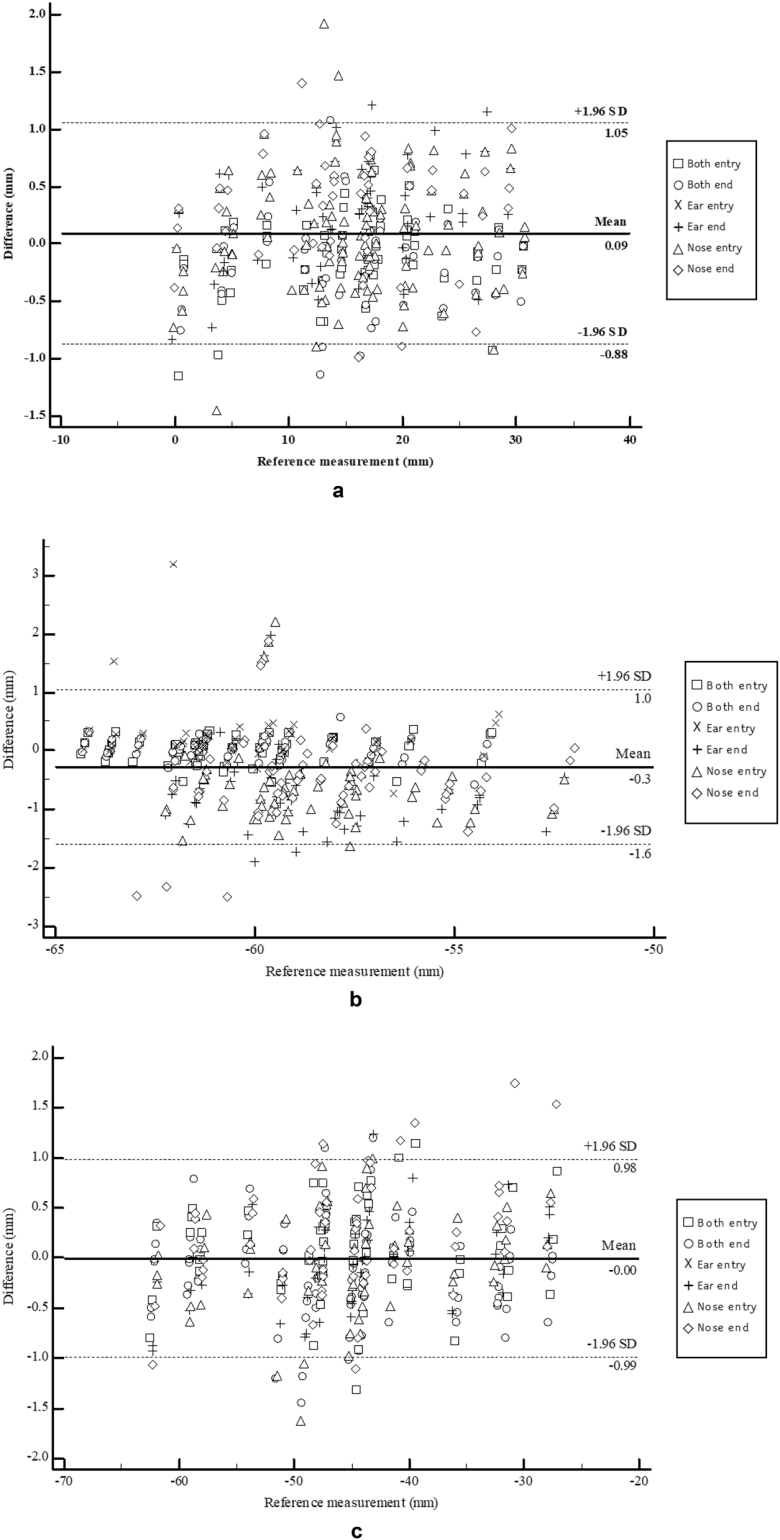
Bland–Altman plot evaluation of the accuracy of the entry point of the skin cancer guide using nose, ear, and both nose and ear as an attached area for X, Y, and Z axes. (**a**) Planned point vs actual point at X-axis. (**b**) planned point vs actual point at Y-axis. (**c**) planned point vs actual point at Z-axis.

**Figure 4:**
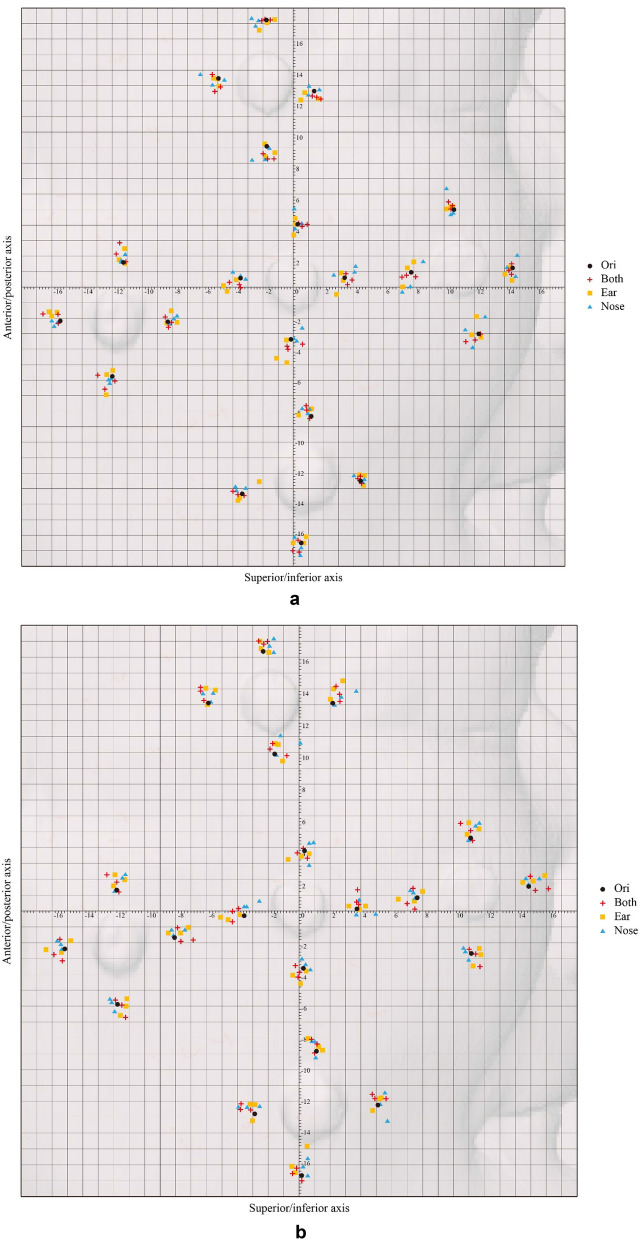
A graphic representation of the planned point and an actual point placement on the skin surface. (**a**) Placement of the entry point. (**b**) Placement of the endpoint.

To evaluate the accuracy of the skin cancer resection guide, we compared the planned and actual insertion points. Three independent researchers inserted 16-cc intravenous (IV) catheters into the in-vitro phantom. A total of 360 measurement points were obtained by measuring the four injection points including the top, bottom, left, and right of the five cancer locations using the attached regions of the skin cancer resection guide including the nose, ear, and a combination of both. Also included the entry and end of each inserted 16-cc IV catheter. After inserting the 16-cc IV catheter into the in-vitro phantom using each attached region of the guide, a total of six CT scans were obtained via the head and neck multi-detector CT (MDCT) scan of the in-vitro phantom. All CT images were segmented into the skin and 16-cc IV catheter and then converted to Stereo Lithography (STL). Each STL was matched using the global registration with manual correction. The differences in planned and actual points were measured using 3-Matics V9 (Fig. 2). The entry point measurement errors [mean ± standard deviation (SD)] of the attached regions of the guide at the nose, ear, and a combination of both were 1.523 ± 0.786 mm, 1.534 ± 0.78 mm, and 1.395 ± 0.712 mm, respectively. The endpoint measurement errors (mean ± SD) of the attached regions of the guide at the nose, ear, and a combination of both were 2.524 ± 1.049 mm, 2.643 ± 0.944 mm, and 2.924 ± 0.947 mm, respectively (Table 1). We created a line connecting the entry points and the endpoints. Vectors were originated from this line. The accuracy of the insertion angle was measured by measuring the cosine similarity between the vector of the planned line and the vector of the inserted line. The cosine similarity of the attached regions of the guide at the nose, ear, and a combination of both were 0.980 ± 0.022, 0.981 ± 0.017, and 0.980 ± 0.024, respectively (Table 1). Repeated measurement analysis of variance (RM- ANOVA) was used to analyze the statistical differences between each attachment area of the guide and the statistical difference between operators. The difference of each attached region of the guide satisfies Mauchly's test of sphericity (Mauchly's W = 0.988, p = 0.486), and there was no significant difference according to the attached region (F = 0.820, p = 0.442). Also, the difference of each operator does not satisfy Mauchly's test of sphericity (Mauchly's W = 0.930, p = 0.015), and Wilks’s lambda value of the multivariate test was taken. There was no interaction between each operator and inserted point (F = 1.962, p = 0.145). The Bland–Altman plot was used to evaluate the accuracy of the entry point of the skin cancer guide using the nose, ear, and a combination of both as attached regions for the X, Y, and Z axes. Each measurement error (mean ± SD) measured in the X, Y, and Z axes was − 0.879 ± 0.483 mm (limit of agreement from − 0.88 to 1.05 mm), − 0.271 ± 0.656 mm (limit of agreement from − 1.6 to 1.0 mm), and − 0.002 ± 0.500 mm (limit of agreement from − 0.99 to 0.98 mm) (Fig. 3). Finally, a graphic representation of the planned point and an actual point placement on the skin surface is illustrated in Fig. 4.

**Table 1:** The distance between the planned and the actual point and the cosine similarity of the vector of the planned line and the actual line.

	Entry point (mm)		Endpoint (mm)		Cosine similarity	
	Mean	SD	Mean	SD	Mean	SD
**Both**						
Top	1.431	0.620	2.894	1.333	0.975	0.016
Bottom	1.185	0.688	3.452	1.333	0.981	0.015
Center	1.413	0.493	2.567	0.749	0.977	0.040
Left	1.649	0.950	2.652	0.775	0.991	0.018
Right	1.297	0.743	3.054	0.671	0.976	0.021
Total	1.395	0.712	2.924	0.947	0.980	0.024
**Ear**						
Top	1.310	0.589	2.635	1.191	0.970	0.024
Bottom	1.498	0.570	2.763	0.853	0.981	0.015
Center	1.820	1.101	2.503	1.030	0.985	0.015
Left	1.427	0.807	2.711	0.673	0.987	0.009
Right	1.614	0.750	2.539	1.034	0.979	0.014
Total	1.534	0.780	2.643	0.944	0.981	0.017
**Nose**						
Top	1.514	0.613	2.649	1.344	0.969	0.028
Bottom	1.302	0.515	2.377	0.805	0.977	0.022
Center	1.564	0.875	2.449	1.283	0.992	0.007
Left	1.093	0.488	2.114	0.909	0.989	0.012
Right	2.143	0.997	3.031	0.723	0.971	0.027
Total	1.523	0.786	2.524	1.049	0.980	0.022

## Discussion

We evaluated the accuracy of the skin cancer resection guide using an in-vitro phantom. Three researchers participated in the experiment, and a total of 360 points were used for analysis due to the attached regions and measurement points, as well as five different cancer locations. The accuracy of the entry point was when the skin cancer resection guide was attached to a combination of the nose and ear, the most reasonable errors were obtained (1.395 ± 0.712 mm). When the skin cancer resection guide was attached to the nose and ear individually, the errors were slightly higher compared to those when the combination of both was used, but these errors were also reasonable (1.523 ± 0.786 mm, 1.534 ± 0.780 mm). Also, the accuracy of the endpoint was when the skin cancer resection guide was attached to the nose, the most reasonable errors were obtained (2.524 ± 1.049 mm). When the skin cancer resection guide was attached to a combination of the nose and ear, and ear individually, the errors were slightly higher compared to the nose, but these errors were also reasonable (2.924 ± 0.947 mm, 2.643 ± 0.944 mm). An attached guide with single anatomy is expected to have a relatively slightly larger error due to relatively weak fixing force compared to an attached guide with two anatomies. To prevent this error, we made a leader line on the guide body pointing to eye. The skin cancer resection guide is designed considering the insertion depth but is relatively inaccurate, because the 16-cc IV catheter is pushed out slightly when it removes the needle, and the needle and guide are removed. Also, when the guide was removed from the in-vitro phantom. It is expected that a 16-cc IV catheter is pushed out slightly. So, the endpoint shows a relatively high error compared to the entry points. When using the skin cancer resection guide in a real operating room, the cancer area is marked by blue dye injection, indicating that it could be applied in practical scenarios. We used RM-ANOVA to confirm that there was no statistical difference between the three attached regions and that there was no statistical difference between operators. However, this study has several limitations. We artificially fabricated and evaluated cancer only between the nose and ears. We were unable to evaluate the accuracy of the skin cancer resection guide in cases where the cancer was in the back of the head or parietal regions. Follow-up studies will be conducted to evaluate improved skin cancer resection guides using the in-vitro phantom for larger cancers and their wider distributions. We used only one FDM printer and one material to evaluate the accuracy of the skin cancer resection guide, which can result in relatively low reproducibility in other environments. The skin was fabricated to be as close as possible to the human skin using silicone^18–20^, but the repulsive force of the actual injection was not reproduced. Therefore, the environment was slightly different from that in actual operating rooms. Silicone materials are developing rapidly, and therefore, in future studies, we will test new silicone materials and use them to make an in-vitro phantom similar to human skin. In conclusion, the skin cancer resection guide could make the skill gap between novice and expert surgeons smaller by accurately marking the surgical plan on the patient's skin through medical imaging and 3D printing technology. Additionally, the incision area could be minimized by providing an accurate area, and the operating time could be reduced. In future studies, it will be possible to extend these surgical guides into the application of various cancers such as osteosarcoma, kidney cancer, and liver cancer.

## Methods

**Figure 5:**
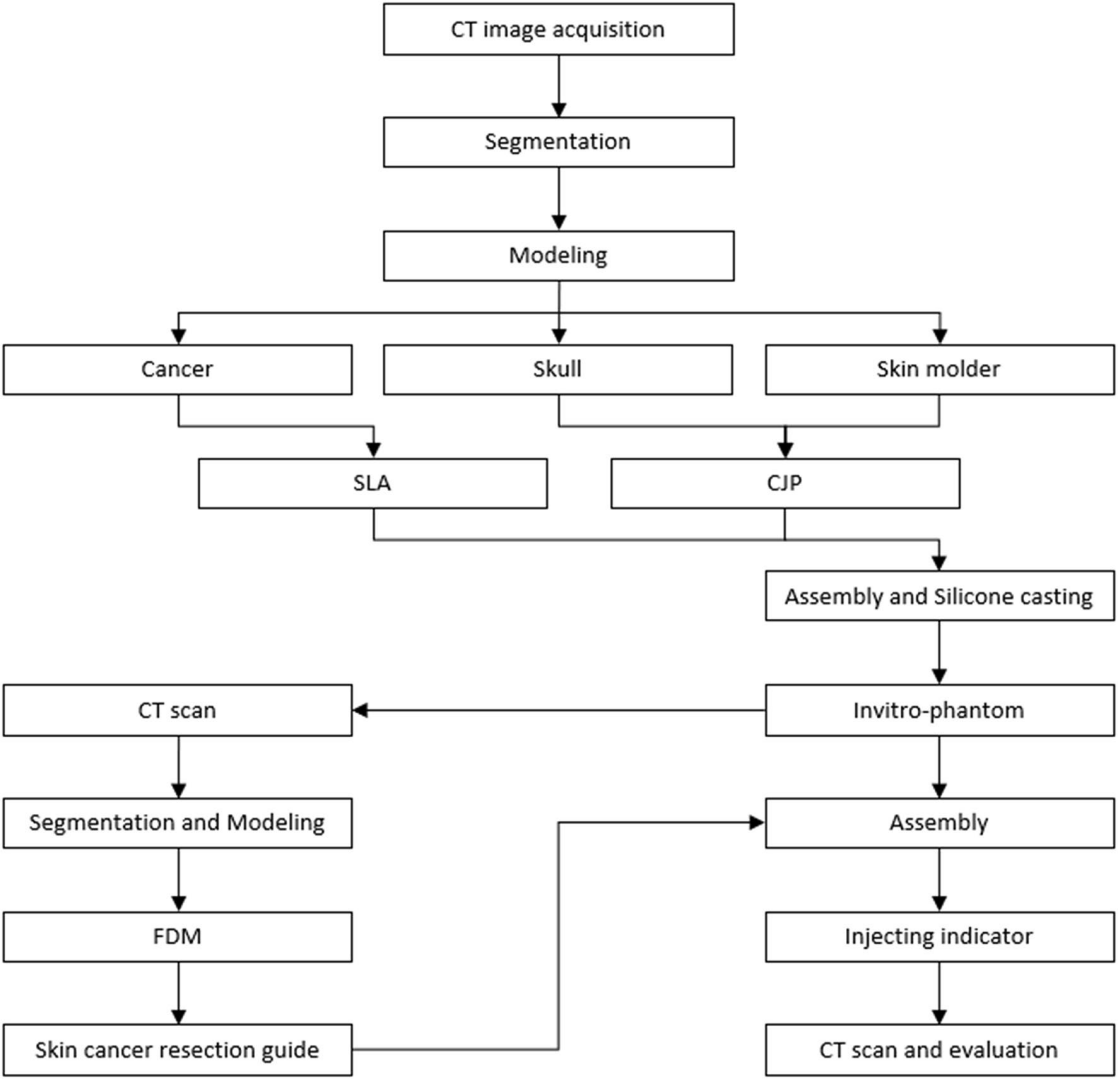
The overall procedure of fabricating and evaluating the skin cancer resection guide and hyper-realistic head in-vitro phantom. *FDM* fused deposition modeling, *CJP* color-jet printing, *SLA* stereolithography apparatus).

3D printing technology is suitable for fabricating various patient-specific phantoms and patient-specific medical devices based on medical images. Silicone molding technology is also required for patient-specific phantom fabrication. Therefore, in this study, we evaluated a patient-specific surgical guide by using an in-vitro phantom using 3D printing technology and silicone molding technology. Various procedures were used to fabricate patient-specific skin cancer resection guides and in-vitro phantoms (Fig. 5). The skin, skull, and cancer were segmented based on medical images obtained from methods such as CT and MR. An in-vitro phantom was designed based on the segmented anatomies and fabricated by various 3D printing technologies and silicon molding technology. The fabricated in-vitro phantom underwent a head and neck MDCT scan to fabricate a patient-specific skin cancer resection guide. The scanned in-vitro phantom CT images were segmented into the skin, skull, and cancer. A safety margin was suggested by the plastic surgeon based on the segmented anatomies. We then designed the skin cancer resection guide using the segmented anatomies and suggested safety margin. We used the guide to insert a 16-cc IV catheter into the phantom, which underwent a head and neck MDCT scan to evaluate the points and angles of insertion.

### Segmentation and modeling of in-vitro phantom

**Figure 6:**
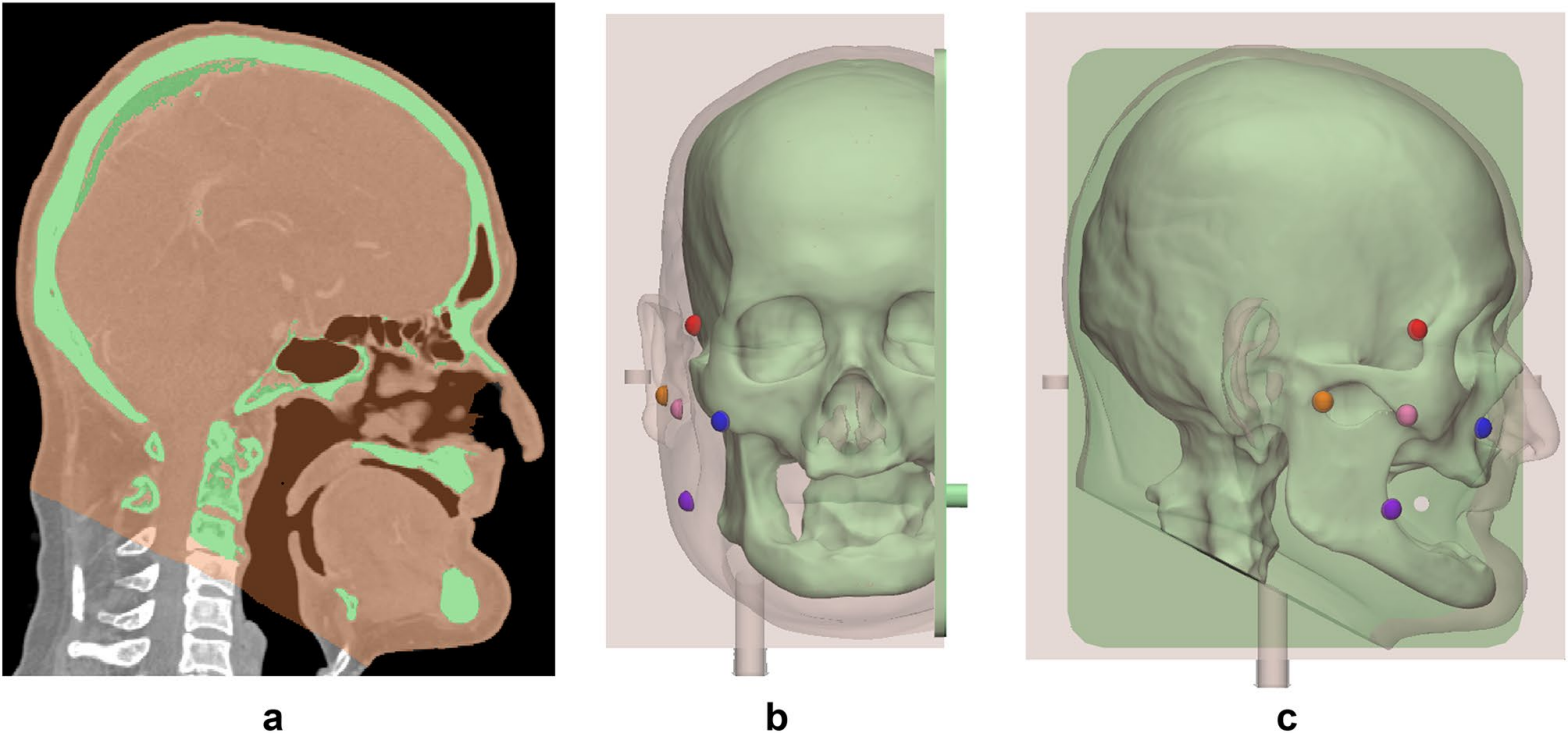
Visualization of segmented anatomies including the skin and skull, and the modeled in-vitro phantom in CT images of a 59-year-old patient with malignant melanoma. (**a**) Sagittal view of segmented anatomies. (**b**) Front view of modeled in-vitro phantom. (**c**) Sagittal view of modeled in-vitro phantom. (skin, peach; skull, light green; cancer top, red; cancer center, pink; cancer bottom, purple; cancer left, orange; cancer right; blue).

A 59-year-old male patient with malignant melanoma underwent a head and neck MDCT scan (SOMATOM Definition Edge, Siemens Healthcare, Erlangen, Germany), which was done at 120 kVp tube voltage and 1-mm slice thickness. The skull and skin were segmented using Mimics v17, a medical image processing software (Materialise Inc., Leuven, Belgium) as shown in Fig. 6a. The skull and skin were segmented using thresholding functions of 289 to 3019 HU and 289 to 3019 HU, respectively, and region growing using manually chosen seeds by an expert. The in-vitro phantom consists of a skull, skin, and five different cancer locations, which were modeled using 3-Matics V9 (Materialise, Belgium). As only the nose and eye areas were required to evaluate the skin cancer resection guides, we did not fabricate the entire face. The cancer model was half-sphere-shaped with a radius of 5 mm. Five different cancers were fabricated using SLA with clear resin and located between the nose and ears. The skull was modeled with a lid to combine with the skin molder, which was divided into top and bottom parts based on the 3D printing output size and separation between the mold and silicone. The skin molder and skull were also produced using CJP with plaster. After combining the top and bottom parts of the skin molder, five different cancers and skulls were inserted into the skin molder, and a hole through which silicone can be injected as well as an air hole were modeled for the stable injection of silicone (Fig. 6b,c).

### Modeling of skin cancer resection guide

**Figure 7:**
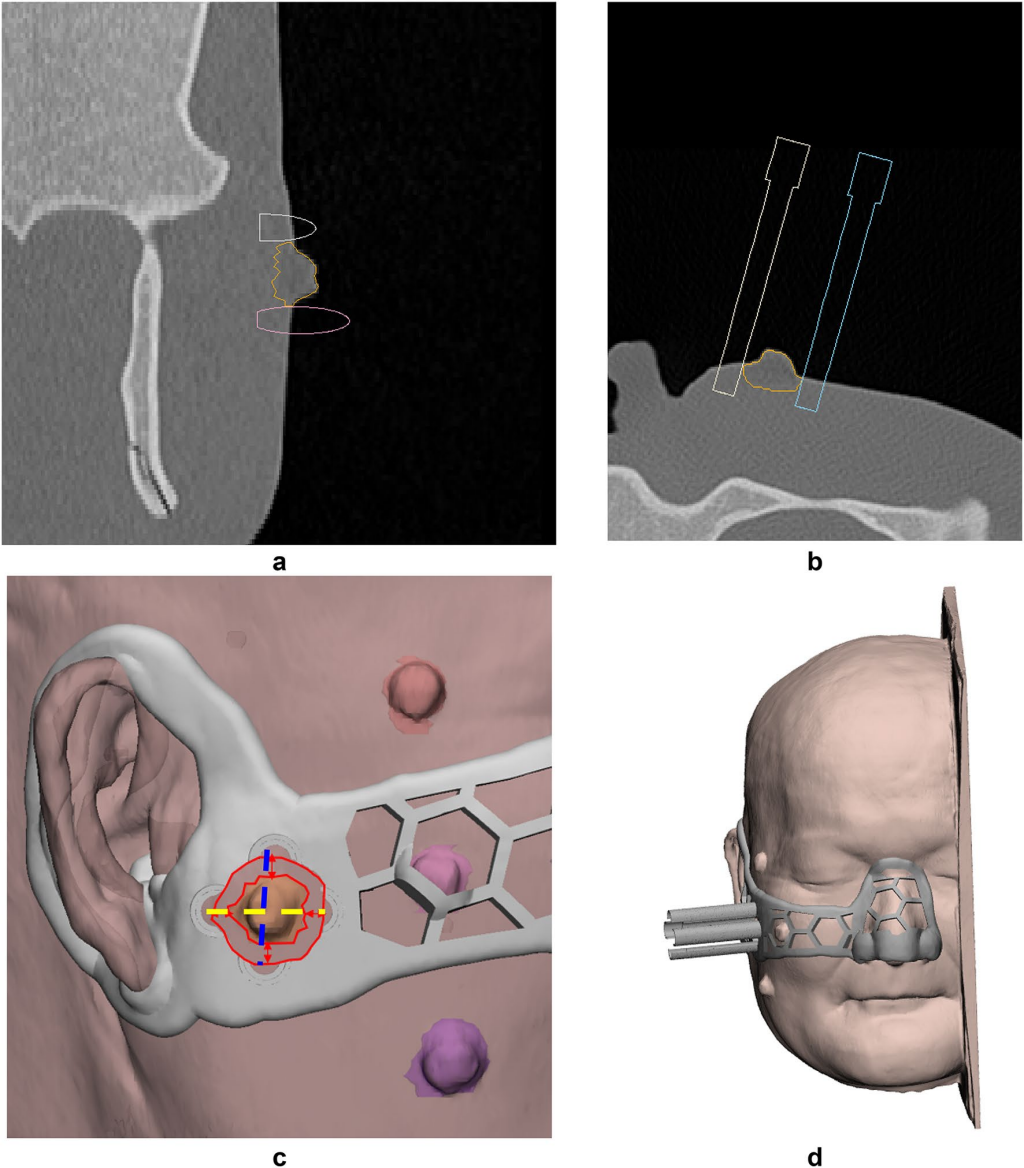
Visualization of scanned in-vitro phantom for evaluating skin cancer resection guide. (**a**) Sagittal view of segmented anatomies and resection point at the blue dotted line in (**c**). (**b**) Axial view of segmented anatomies and intravenous catheter injected to a depth of 7 mm at the yellow dotted line in (**c**). (**c**) Visualization of the skin cancer resection guide and safety resection margin. (**d**) Applying skin cancer resection guide for the attached regions, the combination of nose and ear. (skin, peach; a skull, light green; cancer top, red; cancer center, pink; cancer bottom, purple; cancer left, orange; cancer right, blue).

To fabricate the skin cancer resection guide, the in-vitro phantom was scanned using head and neck MDCT (SIEMENS_S7VA44A, Siemens Healthcare, Erlangen, Germany), at 120 kVp tube voltage and 1-mm slice thickness. The MDCT images were segmented into the skin, skull, and five different cancer locations. The skin cancer resection guide was modeled using the segmented anatomies, and safety margin suggested by a plastic surgeon. The safety resection margin of the lesion was set at 3 mm, and four insertion points including the top, bottom, left, and right of the lesion was designated (Fig. 7c). The nose, ears, and a combination of both were used as the attachments of the guide. The skin cancer resection guide was designed to insert a 16-cc IV catheter to a depth of 7 mm at each point (Fig. 7a,b). To check the position of the guide, we made a leader line pointing to the eye. In addition, each operator was trained to use the leader line. Also, to minimize material consumption, the modeled body was porous (Fig. 7d).

### Evaluation of injection point

Because the attached regions of the guide were the nose, ears, and a combination of both, as well as five different cancer locations, a total of 15 skin cancer resection guides were fabricated. Three researchers independently inserted a 16-cc IV catheter at a total of 360 points using the skin cancer resection guide and scanned these points using head and neck MDCT. Each MDCT image was segmented into the skin and 16-cc IV catheter and then converted into STL, which were matched using the global registration with manual correction. The differences in planned and actual points were measured using 3-Matics V9. In addition, a line was created by connecting the entry points and endpoints.

### Statistical analysis

A Bland–Altman analysis was used to evaluate the planned point and actual point by using Med-calc trial version (MEDCalc Inc, Acacialaan, Belgium), and the RM-ANOVA was used to compare the significant differences among the operators and among the attached regions by using IBM SPSS Statistics, v25.00 (IBM Corp, NewYork, USA).

### Ethical statement

This study was approved by the Institutional Review Board of the Asan Medical Center (IRB No. 2019-0960) and performed according to the principles of the Declaration of Helsinki. It was based on a review of retrospective charts of patients diagnosed with various skin cancers and underwent extensive resection and reconstructive surgery at Asan medical center from February 2016 to July 2018. In addition, the requirement for informed consent was waived by the Institutional Review Board of the Asan Medical Center that approved the study due to retrospective observational studies. We anonymously received CT images of a male patient with malignant melanoma with a 1 mm slice thickness.
