# Author Correction: Evaluation of skin cancer resection guide using hyper‑realistic in‑vitro phantom fabricated by 3D printing

**DOI:** 10.1038/s41598-021-94865-3

**Published:** 2021-08-04

**Authors:** Junhyeok Ock, Taehun Kim, Sangwook Lee, Tae Seong Yang, Minji kim, Wooshik Jeong, Jongwoo Choi, Namkug Kim

**Affiliations:** 1 Department of Convergence Medicine, Asan Medical Institute of Convergence Science and Technology, University of Ulsan College of Medicine, Asan Medical Center, 88 Olympic‑Ro 43‑Gil Songpa‑Gu, Seoul, South Korea; 2 Department of Plastic and Reconstructive Surgery, University of Ulsan College of Medicine, Asan Medical Center, 88 Olympic‑Ro 43‑Gil Songpa‑Gu, Seoul, South Korea; 3 Department of Radiology, University of Ulsan College of Medicine, Asan Medical Center, 388‑1 Pungnap2‑dong, Songpa‑gu, Seoul, South Korea; 4 Department of Plastic and Reconstructive Surgery, Ajou University Hospital, School of Medicine, Suwon, South Korea; 5 ANYMEDI Inc., Seoul, South Korea

Correction to: *Scientific Reports* 10.1038/s41598-021-88287-4, published online 26 April 2021

The original version of this Article contained an error in the spelling of the author Taehun Kim which was incorrectly given as Teahun Kim.

The original Article has been corrected.

